# Plasma and intrapulmonary pharmacokinetics of ceftibuten and ledaborbactam in healthy male and female adults

**DOI:** 10.1128/aac.01950-25

**Published:** 2026-04-20

**Authors:** Keith A. Rodvold, Mark H. Gotfried, Carlos Fernando de Oliveira, Kathryn Lowe, Kamal A. Hamed, Karine Litherland, Marc Engelhardt, Paul C. McGovern

**Affiliations:** 1 University of Illinois Chicago 14681, Chicago, Illinois, USA; 2 Pulmonary Associates, PA, Phoenix, Arizona, USA; 3 Venatorx Pharmaceuticals, Inc., Malvern, Pennsylvania, USA; 4 Basilea Pharmaceutica International Ltd 449688 , Allschwil, Switzerland; Providence Portland Medical Center, Portland, Oregon, USA

**Keywords:** ceftibuten, epithelial lining fluid, ledaborbactam etzadroxil, pharmacokinetics, pulmonary

## Abstract

Ledaborbactam etzadroxil (LED-E) is an oral prodrug of ledaborbactam, a broad-spectrum β-lactamase inhibitor. In combination with ceftibuten (CTB), a third-generation oral cephalosporin, LED-E represents a potential oral β-lactam/β-lactamase inhibitor regimen for treating infections caused by drug-resistant Gram-negative pathogens. This phase 1, single-center, open-label study evaluated intrapulmonary concentrations of ledaborbactam (LED) and CTB in healthy adults. Participants in Group 1 received five oral doses of CTB/LED-E (600 mg/600 mg) every 12 h (q12h), and participants in Group 2 received five oral doses of CTB 600 mg q12h. Following the fifth dose, participants underwent a standardized single bronchoscopy with bronchoalveolar lavage (BAL). Twenty-eight participants in Group 1 and six participants in Group 2 were randomized and dosed. Among participants receiving CTB/LED-E, epithelial lining fluid (ELF) area under the concentration-time curve from 0 to 12 h (AUC_0–12_) for aspirate 1 was 14,867 ng·h/mL for CTB and 14,529 ng·h/mL for LED, and for pooled aspirates 2 + 3 + 4 was 10,486 ng·h/mL and 9,809 ng·h/mL, respectively. ELF-to-unbound plasma AUC ratios were 0.341 (aspirate 1) and 0.239 (pooled aspirates 2 + 3 + 4) for CTB and 1.560 and 1.032, respectively, for LED. Participants receiving CTB alone demonstrated similar lung penetration, indicating no effect of LED on CTB exposure. Both CTB/LED-E and CTB alone were safe and well tolerated; all adverse events were mild or moderate and unrelated to study drug. These results demonstrate pulmonary penetration of CTB and LED and support further evaluation of CTB/LED-E for the treatment of lower respiratory tract bacterial infections caused by susceptible pathogens.

This study is registered with ClinicalTrials.gov as NCT06665555.

## INTRODUCTION

Drug-resistant Gram-negative pathogens pose a critical and escalating public health threat (1, 2). Resistance has been documented to nearly all antibiotic classes, including last-resort agents, such as carbapenems and colistin (3). Globally, antimicrobial resistance has been reported in approximately one in six bacterial infections (Global Antibiotic Resistance Surveillance Report) (4), and in 2021, more than 1.1 million deaths were attributed directly to antimicrobial resistance. Among Gram-negative pathogens, the largest increase in resistance occurred with carbapenems (5). In the United States alone, nearly 3 million infections due to antimicrobial-resistant pathogens were reported in 2019, resulting in more than 35,000 deaths (6).

Despite this growing clinical burden, therapeutic options for drug-resistant Gram-negative infections remain limited. Several parenteral β-lactam/β-lactamase inhibitor combinations have been introduced in recent years; however, the β-lactam armamentarium still lacks an effective oral agent active against these pathogens (1, 2). This limitation restricts options for step-down therapy and outpatient management, underscoring the urgent need for new oral treatments targeting drug-resistant Gram-negative organisms.

Ledaborbactam etzadroxil (LED-E; VNRX-7145) is an oral prodrug of ledaborbactam (LED, VNRX5236), a novel broad-spectrum β-lactamase inhibitor (7). In combination with ceftibuten (CTB), a third-generation oral cephalosporin, LED-E provides an oral β-lactam/β-lactamase inhibitor combination (CTB/LED) for the treatment of infections caused by drug-resistant Gram-negative bacteria. *In vitro*, ledaborbactam restores CTB activity against Enterobacterales with multiple resistance mechanisms, including extended-spectrum β-lactamases (ESBLs) and carbapenemases, such as *Klebsiella pneumoniae* carbapenemase (KPC) and OXA-48 (8–11). *In vivo*, LED enhances activity against CTB-resistant strains and markedly reduces bacterial load in models of drug-resistant Enterobacterales infection (12, 13). Whereas ceftibuten alone is inactive against KPC-producing *K. pneumoniae*, CTB/LED prevents septicemia in murine infection models (14) and achieves bacteriostasis in neutropenic murine thigh models of β-lactamase-producing Enterobacterales (15).

A first-in-human study evaluating single and multiple doses of LED alone and in combination with CTB in healthy adults demonstrated dose-proportional pharmacokinetics (PK) and predominant urinary excretion of LED (84%) (16). A subsequent drug-drug interaction study found a low potential for clinically meaningful interactions between CTB and LED (NCT04877379) (16).

Achieving sufficient epithelial lining fluid (ELF) exposures and pharmacokinetic-pharmacodynamic (PK-PD) target attainment is critical for the treatment of lower respiratory tract infections. Accordingly, this bronchoalveolar lavage (BAL) study characterized the intrapulmonary PK of CTB and LED following co-administration of CTB with LED-E and administration of CTB alone in healthy adult participants.

## RESULTS

A total of 28 participants in Group 1 and six participants in Group 2 were screened, randomized, and dosed. The study initially planned to enroll 25 participants in Group 1; however, one participant withdrew before bronchoscopy due to a non-serious adverse event of anxiety, and two participants had LED sample-processing errors. Consequently, three additional participants were enrolled in Group 1. Overall, the study population was predominantly female (73.5%) and White (58.8%). The mean (standard deviation [SD]) age was 38.8 (10.0) years (Table 1).

**TABLE 1:** Baseline characteristics of study participants

	Group 1 CTB/LED-E^*a*^ (*n* = 28)	Group 2 CTB^*b*^ (*n* = 6)	Overall (*n* = 34)
Age, years^*c*^	38.4 ± 10.6	40.7 ± 6.7	38.8 ± 10.0
Age range, years	21–55	32–49	21–55
Female, *n* (%)	21 (75.0)	4 (66.7)	25 (73.5)
Race, *n* (%)			
White	15 (53.6)	5 (83.3)	20 (58.8)
Black or African American	7 (25.0)	1 (16.7)	8 (23.5)
Asian	2 (7.1)	0	2 (5.9)
Other	4 (14.3)	0	4 (11.8)
Hispanic or Latino, *n* (%)	6 (21.4)	3 (50.0)	9 (26.5)
Body weight, kg^*c*^	79.1 ± 12.8	78.5 ± 12.0	79.0 ± 12.5
Height, cm^*c*^	173.2 ± 9.0	169.7 ± 7.2	172.6 ± 8.7
Body mass index, kg/m^2*c*^	26.3 ± 3.4	27.3 ± 3.7	26.5 ± 3.5

^*a*^ CTB/LED‑E, ceftibuten/ledaborbactam etzadroxil.

^*b*^ CTB, ceftibuten.

^*c*^ Mean ± SD.

### Pharmacokinetics

#### Group 1 (CTB + LED-E)

**Fig 1:**
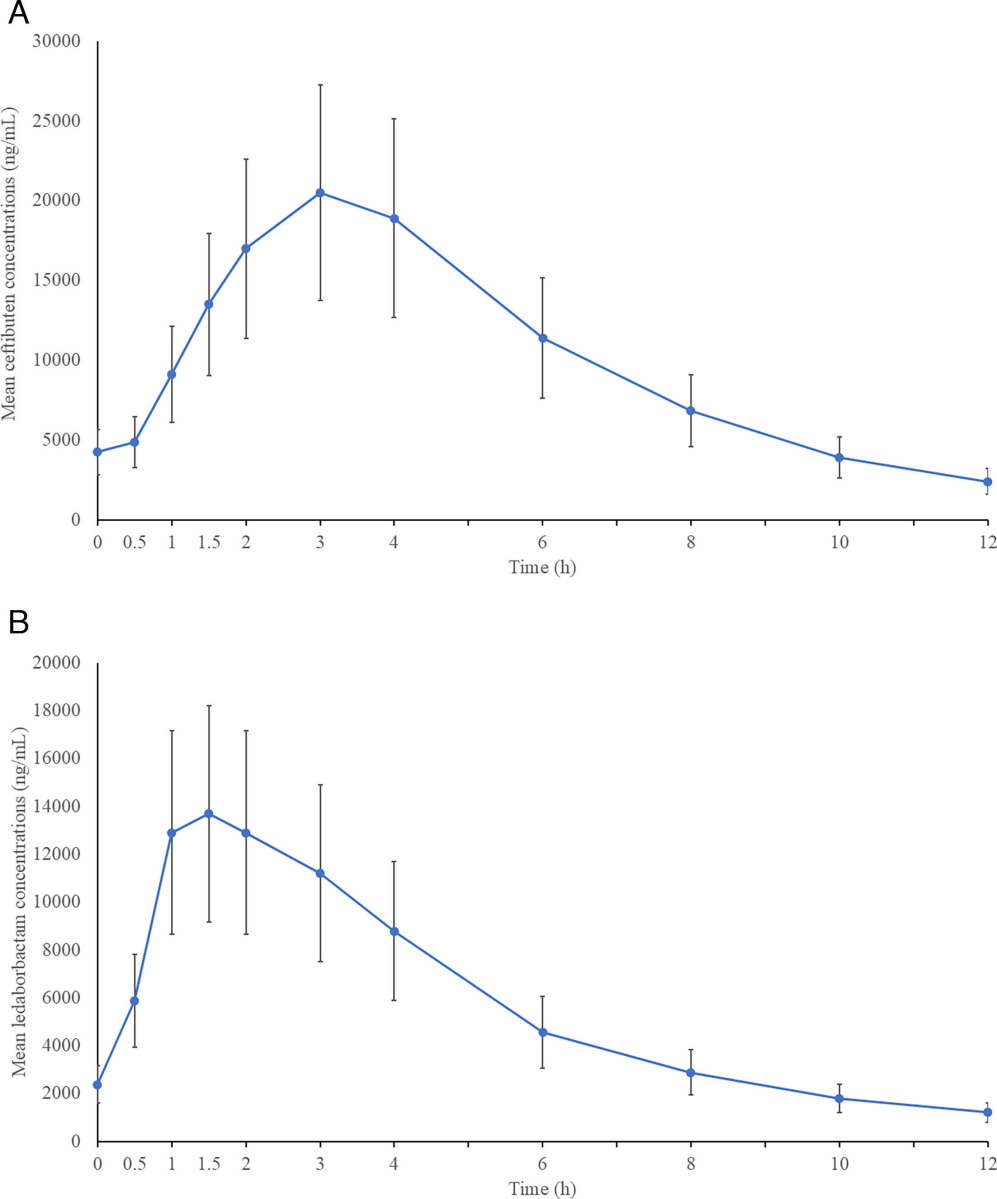
Mean (± SD) plasma concentrations of ceftibuten (**A**) and ledaborbactam (**B**) in Group 1 after the fifth oral dose of ceftibuten 600 mg/ledaborbactam etzadroxil 600 mg every 12 h.

Mean (SD) plasma concentration-time profiles of CTB and LED following the fifth oral dose showed peak concentrations at approximately 3 and 2 h, respectively (Fig. 1; Table 2). Plasma concentrations of both CTB and LED were below the limit of quantitation (BLQ) in all participants prior to the first dose. Plasma concentrations of CTB and LED were measurable in all participants throughout the dosing interval. Mean (SD) PK parameters for CTB and LED are summarized in Table 2.

**TABLE 2:** Mean (± SD) plasma pharmacokinetic parameters^*a*^ for ceftibuten and ledaborbactam after the fifth oral dose of ceftibuten/ledaborbactam etzadroxil (Group 1)

	*C*_max_ (ng/mL)	*T*_max_ (h)	*C*_min_ (ng/mL)	AUC_0–t_ (ng·h/mL)	AUC_0–12_ (ng·h/mL)	CL/F (L/h)	*V*_*z*_/*F* (L)	*t*_1/2_ (h)
Ceftibuten								
All participants^*b*^	22,708 ± 5,317	3.00 (1.98, 5.98)	2,569 ± 1,363	121,794 ± 27,110	121,799 ± 27,105	5.20 ± 1.33	19.41 ± 5.58	2.61 ± 0.51
2-h cohort^*c*^	25,180 ± 4,103	4.00 (2.00, 5.98)	2,542 ± 563	128,315 ± 18,646	128,340 ± 18,634	4.75 ± 0.66	16.50 ± 2.30	2.41 ± 0.14
4-h cohort^*c*^	27,940 ± 3,918	3.00 (2.98, 4.00)	3,404 ± 1,751	152,918 ± 23,932	152,917 ± 23,911	4.00 ± 0.64	15.05 ± 2.98	2.61 ± 0.36
6-h cohort^*c*^	18,840 ± 6,590	3.00 (2.03, 3.98)	2,436 ± 2,252	107,046 ± 30,397	107,042 ± 30,404	6.06 ± 2.01	23.51 ± 8.25	2.79 ± 1.03
8-h cohort^*c*^	19,540 ± 3,014	3.00 (2.00, 4.00)	2,466 ± 841	107,029 ± 17,746	107,031 ± 17,754	5.74 ± 1.03	22.51 ± 3.82	2.74 ± 0.37
12-h cohort^*c*^	22,040 ± 3,357	2.98 (1.98, 4.00)	1,998 ± 822	113,663 ± 18,936	113,663 ± 18,936	5.43 ± 1.13	19.46 ± 4.77	2.48 ± 0.31
Ledaborbactam								
All participants^*b*^	15,796 ± 2,754	1.50 (0.97, 3.98)	1,275 ± 489	70,403 ± 12,963	70,406 ± 12,964	8.78 ± 1.48	35.06 ± 5.81	2.79 ± 0.34
2-h cohort^*c*^	15,440 ± 1,832	1.48 (0.97, 3.98)	1,230 ± 286	66,867 ± 8,544	66,879 ± 8,552	9.09 ± 1.19	35.98 ± 5.92	2.73 ± 0.13
4-h cohort^*c*^	17,920 ± 2,030	1.50 (1.50, 3.00)	1,748 ± 642	82,861 ± 15,474	82,864 ± 15,471	7.42 ± 1.21	32.78 ± 5.81	3.07 ± 0.35
6-h cohort^*c*^	16,560 ± 3,871	1.00 (1.00, 2.00)	957 ± 476	69,420 ± 16,701	69,423 ± 16,699	9.03 ± 2.05	32.09 ± 7.13	2.48 ± 0.28
8-h cohort^*c*^	15,200 ± 2,864	2.98 (1.48, 3.02)	1,212 ± 290	69,166 ± 6,897	69,166 ± 6,913	8.74 ± 0.78	34.05 ± 3.76	2.71 ± 0.31
12-h cohort^*c*^	13,860 ± 1,781	1.00 (0.98, 2.00)	1,258 ± 461	63,700 ± 10,018	63,700 ± 10,018	9.60 ± 1.44	40.42 ± 3.61	2.95 ± 0.34

^*a*^ Data are expressed as mean ± SD except for *T*_max_, which is presented as median (range). AUC_0–12_, area under the plasma concentration-time curve from time 0 to 12 h; AUC_0–t_, area under the plasma concentration-time curve from time 0 to the time of the last quantifiable concentration; CL/F, apparent total plasma clearance; *C*_max_, maximum plasma concentration; *C*_min_, minimum plasma concentration; *t*_1/2_, elimination half-life; *T*_max_, time to *C*_max_; *V*_*z*_/*F*, apparent volume of distribution at terminal phase.

^*b*^ 25 parameter values for each listing.

^*c*^ 5 parameter values for each listing.

**Fig 2:**
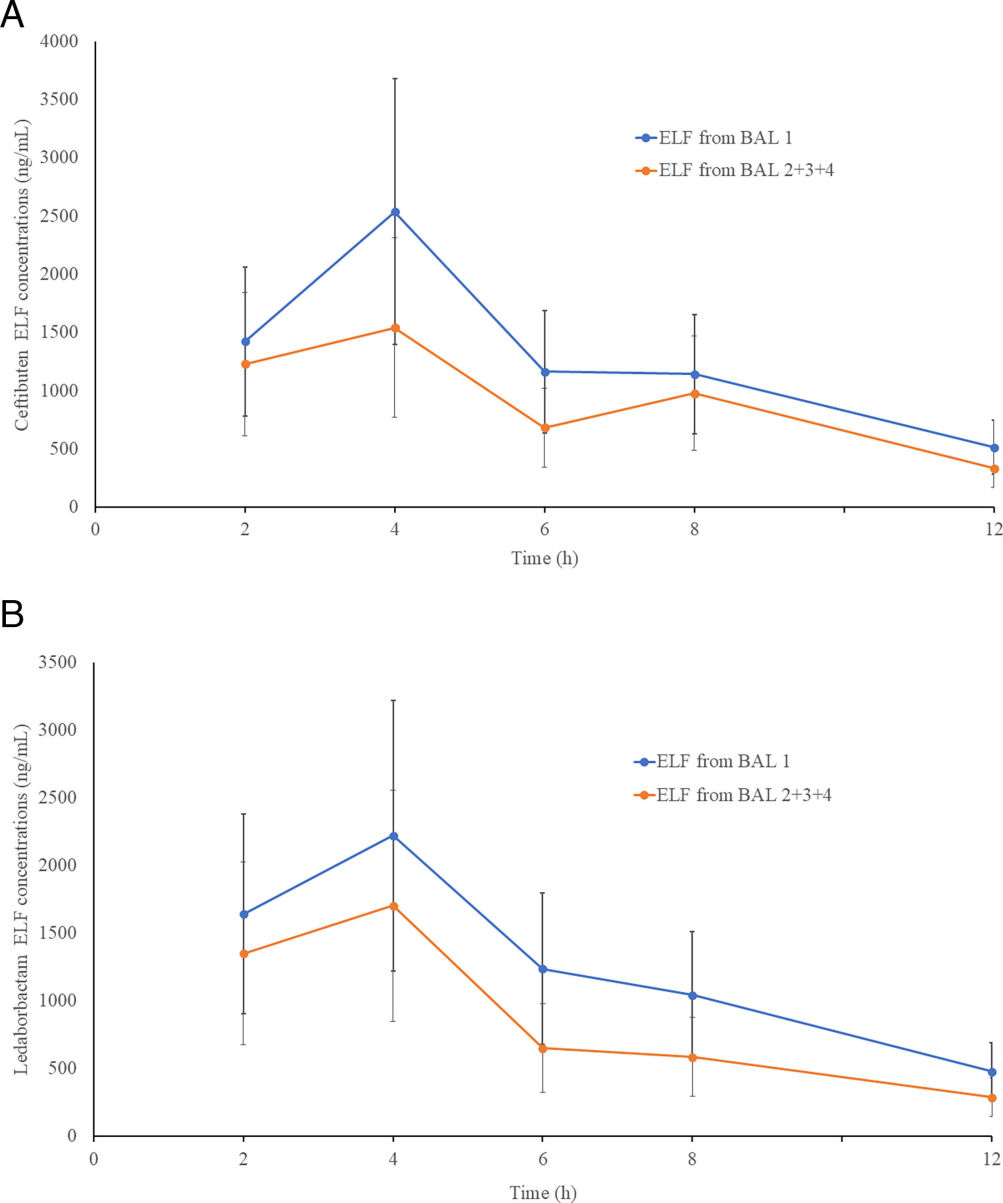
Mean (± SD) ceftibuten (**A**) and ledaborbactam (**B**) ELF concentrations in Group 1 for BAL aspirate 1 and BAL pooled aspirates 2 + 3 + 4 at the scheduled BAL sampling times after the fifth oral dose of ceftibuten 600 mg/ledaborbactam etzadroxil 600 mg every 12 h. BAL, bronchoalveolar lavage; ELF, epithelial lining fluid.

For BAL sampling, mean (SD) plasma and BAL urea concentrations, aspirated BAL fluid volumes, and apparent ELF volumes were obtained for aspirate 1 and pooled aspirates 2 + 3 + 4. Compared with aspirate 1, pooled aspirates 2 +3 + 4 showed higher urea concentrations in BAL and larger apparent ELF volumes (Table 3). The mean (SD) CTB and LED concentrations in ELF across assessed BAL sampling times exhibited peak concentrations at 4 h (Table S1). Across bronchopulmonary sampling times, ELF concentrations for both CTB and LED were higher in aspirate 1 than in pooled aspirates 2 + 3 + 4 (Fig. 2).

**TABLE 3:** Mean (± SD) urea concentrations in plasma and BAL^*a*^, volume of BAL aspirates, and calculated epithelial lining fluid (ELF)

Measurement	Aspirate 1	Pooled aspirates 2 + 3 + 4
Group 1		
Plasma urea concentration (µg/mL)^*b*^	295.9 ± 78.1	295.9 ± 78.1
BAL urea concentration (µg/mL)^*b*^	3.4 ± 2.2	5.0 ± 2.2
BAL volume (mL)^*b*^	22 ± 4	117 ± 15
Apparent ELF volume (mL)^*b*^	0.25 ± 0.14	1.99 ± 0.60
Group 2		
Plasma urea concentration (µg/mL)^*c*^	348.9 ± 82.6	348.9 ± 82.6
BAL urea concentration (µg/mL)^*c*^	2.8 ± 1.7	5.8 ± 3.8
BAL volume (mL)^*c*^	22 ± 6	117 ± 15
Apparent ELF volume (mL)^*c*^	0.15 ± 0.05	1.86 ± 0.54

^*a*^ BAL, bronchoalveolar lavage.

^*b*^ 25 parameter values for each listing.

^*c*^ 6 parameter values for each listing.

Area under the concentration-time curve from 0 to 12 h (AUC_0–12_) values in ELF for aspirate 1 and pooled aspirates 2+3+4 were 14,867 and 10,486 ng·h/mL for CTB and 14,529 and 9,809 ng·h/mL for LED, respectively (Table 4). The corresponding ELF-to-unbound plasma AUC ratios (DPR_ELF/plasma_, drug penetration ratio into ELF) were 0.341 and 0.239 for CTB and 1.560 and 1.032 for LED, respectively. Both ELF concentrations and DPR_ELF/plasma_ values were higher for aspirate 1 than for pooled aspirates 2 + 3 + 4. The mean (SD) ratios of ELF to simultaneous unbound plasma concentrations generally increased from the earliest through the last BAL sampling times (Table S2).

**TABLE 4:** Exposure and penetration ratio for ceftibuten and ledaborbactam in plasma and ELF for BAL aspirates^*a*^ (Group 1)

Parameter	Aspirate 1	Pooled aspirates 2 + 3 + 4
Ceftibuten		
AUC_0–12_ plasma (total)	124,549	125,601
AUC_0–12_ plasma (unbound)^*b*^	43,592	43,960
AUC_0–12_ ELF	14,867	10,486
DPR_ELF/Plasma_	0.341	0.239
*t*_1/2-ELF_ (h)	4.78	4.96
Ledaborbactam		
AUC_0–12_ plasma (total)	62,092	63,361
AUC_0–12_ plasma (unbound)^*b*^	9,314	9,504
AUC_0–12_ ELF	14,529	9,809
DPR_ELF/Plasma_	1.560	1.032
*t*_1/2-ELF_ (h)	4.24	4.88

^*a*^ AUC_0–12_ units expressed as ng·h/mL. Mean concentration values at each BAL sampling time were determined, and data from all sampling times were combined into a single dataset to calculate the AUC_0–12_ value for each matrix. AUC_0–12_, area under the plasma concentration-time curve from time 0 to 12 h; BAL, bronchoalveolar lavage; DPR_ELF/Plasma_, drug penetration ratio into ELF (ratio of AUC_0–12_ in ELF to AUC_0–12_ unbound plasma); ELF, epithelial lining fluid; *t*_1/2_, terminal half-life.

^*b*^ The unbound fraction used for ceftibuten in plasma was 0.35, and the unbound fraction used for ledaborbactam was 0.15.

#### Group 2 (CTB only)

Plasma concentrations of CTB were BLQ in all participants prior to the first dose. Following the fifth dose, the mean (SD) plasma concentrations peaked at approximately 3 h, and the concentration-time profile for CTB alone was similar to that observed in Group 1. CTB plasma concentrations were measurable in all participants, with PK parameters indicating higher concentrations at 4 h than at 12 h (Table S3).

The mean (SD) plasma and BAL urea concentrations, aspirated BAL fluid volumes, and apparent ELF volumes for aspirate 1 and pooled aspirates 2 + 3 + 4 are summarized in Table 3. Consistent with Group 1, pooled aspirates 2 + 3 + 4 showed higher BAL urea concentrations and larger apparent ELF volumes compared with aspirate 1. The mean (SD) total and unbound plasma concentrations, ELF concentrations, and ELF-to-unbound plasma ratios for CTB across BAL sampling times are presented in Table S4. At the 4-h time point, ELF-to-unbound plasma concentration ratios were 0.272 for aspirate 1 and 0.222 for pooled aspirates 2 + 3 + 4, which were comparable to those observed at 4 h in Group 1. At the 12-h time point, only one measurable ELF sample was available. AUC_0 –12_ exposure and penetration ratios were not estimated for Group 2 because only two sampling time points were evaluated, and five of six CTB concentrations at 12 h were BLQ.

### Safety

Three (10.7%) participants in Group 1 experienced at least one treatment-emergent adverse event (TEAE); all events were considered by the investigator to be unrelated to the study drug (Table 5). One (3.6%) participant in Group 1 experienced a moderate, non-serious adverse event of anxiety, assessed as unrelated to study drug, and discontinued the study. No clinically meaningful changes or trends were observed in hematology, blood chemistry, urinalysis, coagulation parameters, vital signs, physical examinations, or electrocardiograms (ECGs). Loose stools were recorded in seven (25.0%) participants overall; however, only two participants in Group 1 (7.1%) and none in Group 2 met the standard definition of diarrhea (≥3 loose stools per day) (17). These occurrences of loose stools did not represent a change from baseline bowel habits and were not classified as adverse events by the investigator.

**TABLE 5:** Incidence of treatment-emergent adverse events

	Group 1 (*n* = 28) *n* (%)	Group 2 (*n* = 6) *n* (%)	Overall (*N* = 34) *n* (%)
Any TEAE^*a*^	3 (10.7)	0	3 (8.8)
Any drug-related TEAE	0	0	0
Serious TEAE	0	0	0
TEAE leading to early withdrawal from study	1 (3.6)	0	1 (2.9)
TEAEs by severity			
Mild	1 (3.6)	0	1 (2.9)
Moderate	2 (7.1)	0	2 (5.9)
Severe	0	0	0
Individual TEAEs			
Anxiety	1 (3.6)	0	1 (2.9)
Dyspepsia	1 (3.6)	0	1 (2.9)
Headache	1 (3.6)	0	1 (2.9)

^*a*^ Treatment-emergent adverse event. Adverse events are coded using MedDRA version 27.0.

## DISCUSSION

Successful treatment of serious respiratory infection requires that systemic antimicrobial agents achieve intrapulmonary drug concentrations that exceed the MIC of the target pathogen. This is particularly critical for patients in intensive care units, in whom altered physiology and severe illness may influence drug distribution (18–22). Although many antimicrobial classes, including β-lactams, fluoroquinolones, and macrolides, achieve acceptable penetration into intrapulmonary tissues, others, such as aminoglycosides, may attain subtherapeutic concentrations (20, 21). For antimicrobials in development for lower respiratory infections, determining intrapulmonary penetration using bronchoscopy with BAL is therefore essential to confirm adequate drug concentrations in ELF (18, 23).

This study evaluated the safety, intrapulmonary PK, and plasma PK of CTB and LED-E in healthy participants. The highest ELF exposures were observed when the first BAL aspirate was used to calculate AUC_0–12_, ELF/plasma AUC_0–12_ ratios, and ELF-to-simultaneous unbound plasma concentration ratios for both CTB and LED. These observations were driven by a marked relative increase in urea concentrations and apparent ELF volumes in pooled aspirates (2 + 3 + 4) compared with the first aspirate. Similar patterns of higher ELF drug concentrations and lower ELF urea concentrations in the first aspirate have been previously reported for cefepime in a dog lung model and for cefepime and taniborbactam in healthy adults (24, 25).

Measured ELF concentrations were assumed to represent unbound drug, as only unbound plasma drug is expected to penetrate pulmonary compartments. CTB concentrations in ELF were lower than both total and unbound plasma concentrations, whereas LED concentrations in ELF were lower than total plasma concentrations but exceeded unbound plasma concentrations. The assumed plasma unbound fraction for LED (15%) was the lowest estimate from a prior phase 1 study (16). Although a higher unbound fraction would proportionally reduce calculated DPR_ELF/plasma_, this would not meaningfully alter the interpretation of the results. Further investigation is needed to define the quantitative PK-PD targets for CTB and LED in a murine lung infection model, to support dose modeling and prediction of intrapulmonary target attainment.

When CTB was administered alone, the 4-h ELF-to-simultaneous unbound plasma concentration ratio was similar to that observed with co-administration of CTB and LED-E, suggesting that co-administration does not adversely affect CTB distribution into ELF.

The plasma PK profiles of CTB and LED were consistent with previous clinical studies (16, 26–29). In a prior single- and multiple-ascending dose study of LED-E, mean *C*_max_ ranged from 4.0 to 26.5 µg/mL following 100–1,000 mg single doses and from 3.7 to 15.8 µg/mL after multiple doses of 75–500 mg (16). LED demonstrated robust penetration into intrapulmonary tissues, and CTB intrapulmonary penetration was consistent with prior observations (30, 31), although these earlier studies did not employ contemporary methods for assessing pulmonary antibiotic penetration.

The study has several limitations. First, the use of the urea method may not provide fully accurate estimates of ELF volume (25). Second, protein binding estimates used to derive unbound plasma concentrations were based on previous studies (32, 33), which were also conducted in healthy participants. Third, only a single 4-h time point for CTB alone was available for intrapulmonary concentrations. However, Group 2 was not designed to develop an independent ELF concentration-time profile for CTB alone, but to assess the potential for intrapulmonary interaction between CTB and LED. Finally, the study’s evaluation of ELF penetration in healthy participants may not fully reflect the conditions of lower respiratory tract infections, where factors, such as tissue permeability and ELF protein composition (and thus drug binding), can differ.

From a safety perspective, headache and diarrhea/loose stools were observed at a low frequency in this study. These events were previously reported with CTB monotherapy at the approved 400 mg once-daily dosage (33). However, no formal comparisons can be made across studies or dosing regimens. Overall, CTB was safe and well tolerated in this study.

In summary, these PK results indicate that both CTB and LED achieve lung penetration, supporting further development considerations of CTB combined with LED-E as a potential treatment for lower respiratory tract bacterial infections caused by susceptible pathogens. The CTB/LED-E combination was safe and well tolerated in healthy adult participants.

## MATERIALS AND METHODS

The study was conducted between November 2024 and March 2025 at Pulmonary Associates (Phoenix, AZ) in accordance with the U.S. Code of Federal Regulations, the ethical principles of the Declaration of Helsinki, Good Clinical Practice guidelines, and International Council for Harmonisation standards. The study protocol was approved by an Institutional Review Board, and written informed consent was obtained from each participant before any study procedures were performed. This study was registered at ClinicalTrials.gov (NCT06665555).

### Study design

This was a phase 1, single-center, open-label study conducted in healthy, non-smoking adult male and female participants. In Group 1, 25 participants received five oral doses of CTB/LED-E (CTB 600 mg/LED-E 600 mg) administered q12h. After the fifth dose, each participant underwent a single standardized bronchoscopy with BAL at one of five post-dose time points: 2, 4, 6, 8, and 12 h (*n* = 5 participants per time point). In Group 2, six additional participants received five oral doses of CTB 600 mg administered q12h. After the fifth dose, each participant underwent one standardized bronchoscopy with BAL at either 4 or 12 h post-dose (*n* = 3 participants per time point).

### Study population

Eligible participants were healthy adults aged 18–55 years, with a body mass index (BMI) ≥18.0 and ≤32 kg/m^2^ and a body weight >50 kg for men and ≥45 kg for women. Participants were required to have a forced expiratory volume in 1 second (FEV_1_) ≥80% of predicted at screening and to have been non-smokers and non-vapers for at least 14days prior to screening. Participants were medically healthy, with no clinically significant abnormalities based on medical history, physical examination, vital signs, 12-lead ECG, and clinical laboratory assessments. Women were required to be non-pregnant, non-lactating, and to use a highly effective method of contraception during the study and for 90 days afterward. Men were required to be abstinent or to use a condom during the study and for 90 days afterward. All bowel movements, including number and consistency, were to be recorded.

Participants were excluded if they had a history of any significant medical condition; a history within 6 months of known or suspected *Clostridioides difficile* infection; or a recent history of alcohol consumption exceeding an average of two standard drinks per day. Participants were also excluded if they had a corrected QT interval using Fridericia’s correction ≥450 ms for males or ≥470 ms for females, any clinically significant cardiac abnormality, or any clinically significant laboratory abnormality. Additional exclusion criteria included a history of hypersensitivity to cephalosporins, penicillins, other β-lactam antibacterial drugs, or any component of the CTB or LED-E formulations.

### Blood sample collection

In Group 1, blood samples for measurement of CTB and LED plasma concentrations were collected at pre-dose (0 h) and 0.5, 1, 1.5, 2, 3, 4, 6, 8, 10, and 12 h following the fifth oral dose of CTB/LED-E. An additional pre-dose blood sample (within 2 h prior to dosing) was collected before the administration of the first CTB/LED-E dose. Each participant in Group1 underwent a single standardized bronchoscopy with BAL at one of five post-fifth-dose time points: 2, 4, 6, 8, or 12 h. In Group 2, participants received five oral doses of CTB 600mg administered q12h. Blood samples for measurement of CTB plasma concentrations were collected at pre-dose (0 h) and at 0.5, 1, 1.5, 2, 3, 4, 6, 8, 10, and 12 h following the fifth dose. An additional pre-dose blood sample (within 2 h prior to dosing) was collected before administration of the first dose. Each participant underwent a single standardized bronchoscopy with BAL at either 4 h (*n* = 3) or 12 h (*n* = 3) after the fifth CTB dose.

### Bronchoscopy and BAL

Standardized bronchoscopy and BAL procedures have been described previously (25, 34–36). Four 50 mL aliquots of sterile saline were sequentially instilled into the targeted lung segment, with each aliquot immediately aspirated and placed on ice as a separate sample. The time of each instillation, the time of sample collection, and the recovered volume were recorded. The first 50 mL aspirate was collected separately (aspirate 1), while the second through the fourth aspirates were pooled (pooled aspirates 2 + 3 + 4). A blood sample for urea determination was collected at the start of the second instillation. Aliquots of BAL fluid were obtained from aspirate 1 and the pooled aspirates 2 + 3 + 4 sample for urea measurement.

### Determination of plasma and BAL fluid concentrations

Plasma and BAL samples were assayed for LED-E, LED, and CTB using validated bioanalytical methods. Human acidified sodium fluoride-potassium oxalate plasma samples were analyzed for LED-E and LED using liquid chromatography–tandem mass spectrometry (LC-MS/MS). Calibration ranges were 0.500–500 ng/mL for LED-E and 10.0–10,000 ng/mL for LED, based on the analysis of 0.100 mL of acidified plasma. CTB plasma concentrations were determined using LC-MS/MS methods that met all validation criteria for accuracy, precision, and bias. Plasma samples were analyzed using a calibration range of 0.150–75.0 µg/mL, based on 0.100 mL of mixed matrix plasma. The lower limit of quantitation was 150 ng/mL and 5 ng/mL for CTB in plasma and BAL, respectively; 10 ng/mL and 5 ng/mL for LED in plasma and BAL, respectively; and 100 mg/mL and 0.2 µg/mL for urea in plasma and BAL, respectively. Plasma and BAL aspirate samples were shipped to Syneos Health (Princeton, NJ) for determination of CTB and LED concentrations, and to Keystone Laboratories (North Wales, PA) for determination of urea concentrations in plasma and BAL.

### Determination of urea concentration

Urea concentration in plasma and BAL fluid supernatants was measured using a validated LC-MS/MS method developed by Keystone Bioanalytical (North Wales, PA). The calibration curve for the plasma urea assay was linear over a range of 100–3,000 µg/mL. Inter-assay precision (percent coefficient of variation [%CV]) and accuracy (percent relative error [%RE]) for plasma urea ranged from 1.98% to 3.37% and −8.17% to 4.13%, respectively. Intra-assay precision and accuracy ranged from 0.56% to 5.60% and −10.21% to 8.54%, respectively. For BAL fluid, the calibration curve was linear over a range of 0.2–10 µg/mL. Inter-assay precision and accuracy ranged from 6.40% to 9.77% and −3.22% to 1.47%, respectively. Intra-assay precision and accuracy ranged from 0.96% to 12.08% and −11.30% to 9.15%, respectively.

### Pharmacokinetic analysis

Noncompartmental PK analysis was performed using Phoenix WinNonlin (version 8.1 or higher; Certara, Princeton, NJ). Maximum plasma concentration (*C*_max_), time to *C*_max_ (*T*_max_), and minimum plasma concentration (*C*_min_) were determined directly from the observed plasma concentration-time profiles following the fifth oral dose of CTB/LED-E. *C*_min_ was defined as the lowest plasma concentration within the 12 h dosing interval and after the fifth dose. The area under the plasma concentration-time curve (AUC) for the fifth dose was calculated using the linear-log trapezoidal method in Phoenix WinNonLin (version 8.5; Certara, Princeton, NJ). AUC values were determined from time 0 to the last quantifiable concentration (AUC_0–last_) and from time 0 to 12 h (AUC_0–12_). Plasma clearance (CL/F) and apparent volume of distribution at steady state (*V*_*z*_/*F*) were also calculated. The elimination rate constant (*K*_el_) was estimated by linear regression of the terminal phase, and elimination half-life (*t*_1/2_) was calculated as *t*_1/2_ = 0.693/*K*_el_. Pre-dose plasma concentrations of CTB and LED BLQ were treated as zero. Post-dose plasma and ELF concentrations BLQ were treated as missing values for all PK calculations.

### Calculations of ELF volume and antibiotic concentrations in ELF

Estimates of ELF volume and drug concentrations were derived from BAL supernatants collected after the first instillation (aspirate 1) and from the pooled second, third, and fourth instillations (pooled aspirates 2 + 3 + 4). Concentrations of CTB and LED in ELF (ABX_ELF_) were calculated as follows:


$$ABX_{ELF}=ABX_{BAL}\times (V_{BAL}/V_{ELF})$$

where *ABX*_*BAL*_ is the measured drug concentration in BAL fluid, *V*_*BAL*_ is the volume of recovered BAL fluid, and *V*_*ELF*_ is the apparent volume of ELF sampled. *V*_*ELF*_ was calculated using urea as an endogenous marker according to the following equation:


$$V_{ELF}=V_{BAL}\times Urea_{BAL}/Urea_{P}$$

where *Urea*_*BAL*_ and *Urea*_*P*_ represent the urea concentrations in BAL fluid and plasma, respectively (37).

Arithmetic mean ELF concentrations of CTB and LED at each BAL sampling time (e.g., 2, 4, 6, 8, and 12 h) were used to estimate the AUC_0–12_ for both plasma and ELF. The elimination rate constant from ELF (*K*_el-ELF_) was determined by linear regression, and the elimination half-life (*t*_1/2-ELF_) from ELF was calculated as *t*_1/2-ELF_ = 0.693/*K*_el-ELF_. AUC_0–12_ for aspirate 1 and pooled aspirates 2 + 3 + 4 was calculated using the linear-log trapezoidal method in Phoenix WinNonLin. The 12-h ELF concentration also served as the time zero value when calculating AUC_0–12_. DPR_ELF/Plasma_ was calculated as the ratio of AUC_0–12_ in ELF to AUC_0–12_ in unbound plasma. Additionally, concentration ratios of ELF to the simultaneous unbound plasma concentrations were calculated for each participant and summarized by group at each sampling time. The unbound fractions used for plasma CTB and LED were based on previous studies and were 0.35 and 0.15, respectively (32, 33).

### Safety assessments

Participants were continuously monitored during bronchoscopy. Vital signs, including blood pressure, heart rate, respiratory rate, and temperature, were recorded prior to bronchoscopy and at 15 min and 2–4 h after the procedure. Additional safety assessments included physical examinations, clinical laboratory evaluations (hematology, blood chemistry, and urinalysis), 12-lead ECG, and monitoring of adverse events.

### Statistical analysis

This was primarily a descriptive study evaluating CTB and LED concentrations in plasma and ELF. No formal statistical hypothesis testing was performed. The sample size of 25 participants in Group 1 (five participants per time point across five sampling times) and six participants in Group 2 (three participants per time point across two sampling times) was selected to provide sufficient PK data following co-administration of CTB and LED-E and administration of CTB alone, respectively. The overall sample size was deemed adequate to evaluate the safety of CTB and CTB/LED-E in healthy adult participants.

## SUPPLEMENTAL MATERIAL

Supplemental material aac.01950-25-s0001.pdf Tables S1 to S4.

ASM does not own the copyrights to Supplemental Material that may be linked to, or accessed through, an article. The authors have granted ASM a non-exclusive, world-wide license to publish the Supplemental Material files. Please contact the corresponding author directly for reuse.
