# Supplementary material for: Plasma and intrapulmonary pharmacokinetics of ceftibuten and ledaborbactam in healthy male and female adults
Source: Antimicrob Agents Chemother. 2026 Apr 20;70(6):e01950-25. doi: 10.1128/aac.01950-25 (PMC13231890; doi:10.1128/aac.01950-25)
Supplement: Supplemental material — Tables S1 to S4. [file aac.01950-25-s0001.pdf]

Supplemental Material

Supplemental Table 1 Mean (± SD) ceftibuten and ledaborbactam concentrations in ELF[a] for BAL[b] aspirates at assessed BAL sampling times (Group 1)

BAL sampling time	Aspirate 1 (ng/mL)	Pooled aspirates 2+3+4 (ng/mL)
**Ceftibuten**		
2-h	1423 ± 1141[c]	1228 ± 826[c]
4-h	2536 ± 1246[c]	1541 ± 531[c]
6-h	1162 ± 370[d]	682 ± 336[e]
8-h	1142 ± 663[f]	978 ± 281[g]
12-h	515 ± 80[g]	335 ± 51[g]
**Ledaborbactam**		
2-h	1642 ± 858[c]	1352 ± 338[c]
4-h	2221 ± 1094[c]	1704 ± 1122[c]
6-h	1239 ± 367[d]	653 ± 247[e]
8-h	1044 ± 478[f]	587 ± 82[f]
12-h	479 ± 159[f]	289 ± 2[h]

[a]Epithelial lining fluid.
[b]Bronchoalveolar lavage.
[c]5 concentration values.
[d]6 concentration values (1 aspirate was below the limit of quantitation [BLQ]).
[e]7 concentration values.
[f]4 concentration values (1 aspirate was BLQ).
[g]3 concentration values (2 aspirates were BLQ).
[h]2 concentration values (3 aspirates were BLQ).

Supplemental Table 2. Mean (± SD) ceftibuten and ledaborbactam concentration ratios in ELF[a] to unbound plasma for BAL[b] aspirates at assessed BAL sampling times (Group 1)

BAL sampling time	Aspirate 1 (ng/mL)	Pooled aspirates 2+3+4 (ng/mL)
**Ceftibuten**		
2-h	0.245 ± 0.112[c]	0.205 ± 0.058[c]
4-h	0.278 ± 0.099[c]	0.174 ± 0.056[c]
6-h	0.366 ± 0.044[d]	0.205 ± 0.068[e]
8-h	0.536 ± 0.379[f]	0.456 ± 0.200[g]
12-h	0.660 ± 0.194[g]	0.416 ± 0.083[g]
**Ledaborbactam**		
2-h	0.997 ± 0.637[c]	0.796 ± 0.229[c]
4-h	1.410 ± 0.257[c]	1.045 ± 0.409[c]
6-h	2.347 ± 0.497[d]	1.225 ± 0.363[e]
8-h	2.481 ± 1.611[f]	1.359 ± 0.487[f]
12-h	2.441 ± 0.609[f]	1.174 ± 0.308[h]

[a]Epithelial lining fluid.
[b]Bronchoalveolar lavage.
[c]5 ratio values.
[d]6 ratio values (1 aspirate was below the limit of quantitation [BLQ]).
[e]7 ratio values.
[f]4 ratio values (1 aspirate was BLQ).
[g]3 ratio values (2 aspirates were BLQ).
[h]2 ratio values (3 aspirates were BLQ).

Supplemental Table 3 Noncompartmental PK parameters[a] of ceftibuten in plasma after the fifth oral dose of ceftibuten 600 mg every 12 h (Group 2)

	**C_{max} (ng/mL)**	**T_{max} (hours)**	**C_{min} (ng/mL)**	**AUC_{0-t} (ng•h/mL)**	**AUC_{0-12} (ng•h/mL)**	**CL/F (L/h)**	**V_Z/F (L)**	**$t_{1/2}$ (h)**
All participants[b]	21017 ± 9828	2.51 (2.00, 3.00)	2017 ± 1490	110974 ± 46546	110962 ± 46516	6.12 ± 2.07	21.11 ± 7.20	2.40 ± 0.19
4-h cohort[c]	26167 ± 12662	2.00 (2.00, 3.00)	2793 ± 1924	137965 ± 56430	137941 ± 56385	5.07 ± 2.66	18.60 ± 10.16	2.53 ± 0.10
12-h cohort[c]	15867 ± 1266	3.00 (2.02, 3.00)	1240 ± 200	83984 ± 6819	83984 ± 6819	7.17 ± 0.57	23.63 ± 2.73	2.28 ± 0.19

[a]Data are expressed as mean ± SD except for T_{max}, which is presented as median (range). AUC0_{-12}, area under the plasma concentration-time curve from time 0 to 12 h; AUC_{0-t}, area under the plasma concentration-time curve from time 0 to the time of the last quantifiable concentration; CL/F, apparent total plasma clearance; C_{max}, maximum plasma concentration; C_{min}, minimum plasma concentration; $t_{1/2}$, elimination half-life; T_{max}, time to C_{max}; Vz/F, apparent volume of distribution at terminal phase.
[b]6 parameter values for each listing.
[c]3 parameter values for each listing.

Supplemental Table 4. Mean (± SD) total and unbound ceftibuten concentrations in plasma, ELF[a] concentration, and concentration ratio of ELF-to-unbound plasma at assessed BAL[b] sampling times and BAL aspirates (Group 2)

Measurement	4-h BAL sampling time		12-h BAL sampling time	
	Aspirate 1	Pooled aspirates 2+3+4	Aspirate 1	Pooled aspirates 2+3+4
Ceftibuten total concentration (ng/mL)[c]	19633 ± 7305	19633 ± 7305	1240 ± 200	1240 ± 200
Ceftibuten unbound concentration (ng/mL)[c]	6872 ± 2557	6872 ± 2557	434 ± 70	434 ± 70
ELF concentration (ng/mL)	1992 ± 1251[c]	1645 ± 1310[c]	BLQ[d]	249.46[e]
Concentration ratio of ELF-to-unbound	0.272 ± 0.102	0.222 ± 0.128	Not determined	0.495[e]

[a]Epithelial lining fluid.
[b]Bronchoalveolar lavage.
[c]3 parameter values for each listing.
[d]BAL samples were reported as below the limit of qunatitation (BLQ) for all 3 participants.
[e]1 parameter value; BAL fluid samples were reported as BLQ for 2 participants.
